# The R2R3-MYB transcription factor GhMYB1a regulates flavonol and anthocyanin accumulation in *Gerbera hybrida*

**DOI:** 10.1038/s41438-020-0296-2

**Published:** 2020-05-20

**Authors:** Chunmei Zhong, Yi Tang, Bin Pang, Xukun Li, Yuping Yang, Jing Deng, Chengyong Feng, Lingfei Li, Guiping Ren, Yaqin Wang, Jianzong Peng, Shulan Sun, Shan Liang, Xiaojing Wang

**Affiliations:** 1 College of Forestry and Landscape Architecture; Key Laboratory of Energy Plants Resource and Utilization, Ministry of Agriculture, P.R. China, South China Agricultural University, Guangzhou, 510642 China; 2 Guangdong Provincial Key Laboratory of Biotechnology for Plant Development, School of Life Sciences, South China Normal University, Guangzhou, 510631 China; 3 Key Laboratory of Plant Resources and Beijing Botanical Garden, Institute of Botany, Chinese Academy of Sciences, Beijing, 100093 China; 4 Key Laboratory of Southern Subtropical Plant Diversity, Fairy Lake Botanical Garden, Shenzhen & Chinese Academy of Sciences, Shenzhen, Guangdong 518004 China

**Keywords:** Plant physiology, Plant morphogenesis

## Abstract

Anthocyanins and flavonols have vital roles in flower coloration, plant development, and defense. Because anthocyanins and flavonols share the same subcellular localization and common biosynthetic substrates, these pathways may compete for substrates. However, the mechanism regulating this potential competition remains unclear. Here, we identified GhMYB1a, an R2R3-MYB transcription factor involved in the regulation of anthocyanin and flavonol accumulation in *gerbera* (*Gerbera* *hybrida*). GhMYB1a shares high sequence similarity with that of other characterized regulators of flavonol biosynthesis. In addition, GhMYB1a is also phylogenetically grouped with these proteins. The overexpression of *GhMYB1a* in *gerbera* and tobacco (*Nicotiana* *tabacum*) resulted in decreased anthocyanin accumulation and increased accumulation of flavonols by upregulating the structural genes involved in flavonol biosynthesis. We further found that GhMYB1a functions as a homodimer instead of interacting with basic helix-loop-helix cofactors. These results suggest that GhMYB1a is involved in regulating the anthocyanin and flavonol metabolic pathways through precise regulation of gene expression. The functional characterization of *GhMYB1a* provides insight into the biosynthesis and regulation of flavonols and anthocyanins.

## Introduction

Flavonoids, especially anthocyanins, are the major orange, red, purple, and blue pigments in flowering plants^1^. In ornamental plants, flower color is the most important horticultural characteristic influencing their commercial value. Recent advances in genetic modification have led to overcoming the limitations of traditional breeding and have substantially increased the amount of novel colors; this is especially true for the introduction of new genes such as structural or regulatory genes of the flavonoid biosynthetic pathway in ornamental plants^2,3^. Generally, rather than modifying a single enzymatic step of the flavonoid biosynthetic pathway, metabolic flux can be better controlled by coordinated regulation of key regulatory proteins in multiple steps^4,5^. However, the candidate regulatory proteins and their target genes need to be functionally characterized in detail.

Nearly all of the structural genes of the flavonoid biosynthetic pathway isolated and characterized to date are conserved in higher plants^6–9^. Chalcone synthase (CHS), which catalyzes the first step of flavonoid biosynthesis and produces naringenin chalcone^7,10^, is known as a gatekeeper in the regulation of flavonoid biosynthesis. After catalysis by flavanone-3-hydroxylase (F3H), the product dihydrokaempferol is subsequently further converted to flavonols, anthocyanins, or their derivatives. Flavonol synthase (FLS) synthesizes kaempferol-type flavonols. Another quercetin-type flavonol is also synthesized by FLS from dihydroquercetin, which is produced by flavonoid-3′-hydroxylase (F3′H)^11^. Both dihydroflavonol reductase (DFR) and anthocyanidin synthase (ANS) are responsible for the synthesis of anthocyanins^7,12^. In addition, anthocyanidin 3-*O*-glycosyltransferase (3GT) has an important role in the stability of anthocyanins in the cellular environment^13^.

Regulatory proteins are needed for the activation or suppression of flavonoid biosynthesis. Transcriptional regulators of the anthocyanin biosynthetic pathway, including R2R3-MYB proteins, basic helix-loop-helix (bHLH) proteins, and WD-repeat (WDR) proteins, generally form a ternary complex^8,14,15^. R2R3-MYB proteins directly bind to the promoters of biosynthetic genes^6,16^ or to genes encoding bHLH regulators and activate their gene expression^17^. Most R2R3-MYB transcription factors depend on cofactors, such as WDR and bHLH proteins, for regulating flavonoid biosynthesis (except for flavonol synthesis)^14,18,19^. Their combinations and interactions determine a set of genes expressed^10,20,21^. Notably, in the control of the flavonol biosynthetic pathway, the members of subgroup seven of the R2R3-MYB family act independently of bHLH cofactors^22–25^. The first R2R3-MYB transcription factors characterized to regulate flavonol biosynthesis were identified in *Arabidopsis thaliana*, including AtMYB12/PFG1, AtMYB11/PFG2, and AtMYB111/PFG3^11,23^. These factors function independently of bHLH cofactors and individually activate a group of flavonol biosynthetic genes, including *CHS*, *CHI*, *F3H*, and *FLS*^11,23^. The expression pattern of *VvMYBF1* characterized in *Vitis vinifera* is highly correlated with that of *VvFLS1* and subsequently affects flavonol accumulation^26^. The heterologous expression of *GtMYBP3* and *GtMYBP4* characterized in *Gentiana trifloral* increases the accumulation of flavonol and activates the expression of flavonol biosynthesis genes in tobacco (*Nicotiana tabacum*) and Arabidopsis^27^. Although extensive efforts have been made to reveal the functions and regulatory molecular mechanisms of MYB transcription factors, some questions remain, such as which R2R3-MYB transcription factors are involved and how do they regulate the accumulation of anthocyanins and flavonols in plant development.

*Gerbera* is an important floriculture crop species used worldwide as a cut flower. Owing to its diverse flower coloration and complex floral organs, *gerbera* is also an ideal model species for studying flower coloration. However, only the R2R3-MYB transcription factor GhMYB10 has been characterized from *gerbera* to date. Overexpression of *GhMYB10* in transgenic tobacco and *gerbera* plants strongly increases anthocyanin accumulation^28,29^. Therefore, the molecular mechanisms and transcription factors controlling flower coloration in *gerbera* remain unknown. In this paper, we report an in-depth characterization of an R2R3-type MYB transcription factor, GhMYB1a, a putative regulator of flavonol biosynthesis. Overexpression of *GhMYB1a* in tobacco or excised petals of *gerbera* resulted in enhanced flavonol content and reduced anthocyanin accumulation through upregulation of the flavonol biosynthetic genes *CHS*, *F3H*, and *FLS*. The inverse correlation between anthocyanin and flavonol levels in *GhMYB1a*-overexpressing lines probably reflects competition between these two branches of flavonoid metabolites. Analysis of protein–protein interactions involving GhMYB1a suggests that this protein might regulate the expression of downstream genes by forming a homodimer. Our functional characterization of GhMYB1a improves our understanding of the regulatory mechanisms underlying the accumulation of anthocyanin and flavonol biosynthesis in *gerbera* and reveals a new candidate gene for genetic manipulation to generate new cultivars.

## Results

### Isolation and characterization of the MYB transcription factor GhMYB1a

**Fig. 1:**
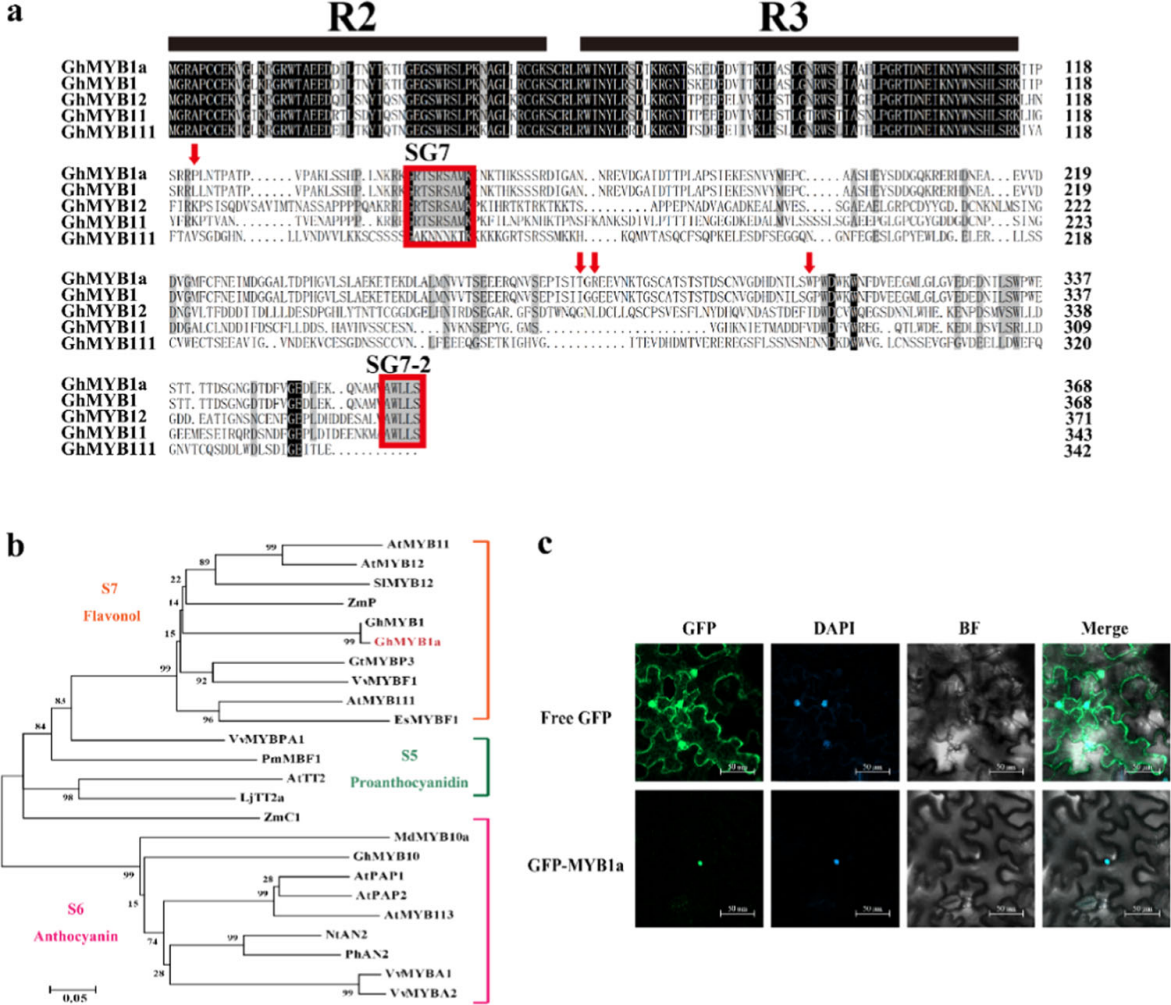
Sequence characteristics of GhMYB1a. **a** Alignment of the protein sequence of GhMYB1a with selected homologs. The alignment was performed using DNAMAN software. The amino acids with 100% identity are shown with a black background, and those with ≥75% identity are shown in gray. The characteristic R2/R3 domains and SG7-1/SG7-2 motifs are shown with black lines and red frames, respectively. The four amino-acid differences between GhMYB1a and GMYB1 are shown with red arrows. **b** Phylogenetic analysis of GhMYB1a and selected R2R3-MYB transcription factors from other plant species. The phylogenetic tree was generated using the neighbor-joining method by MEGA software. The numerals next to the branch nodes indicate bootstrap values from 1000 replications. The bar indicates an evolutionary distance of 0.05%. GhMYB1a is marked in red. The functions of most of the R2R3-MYB transcription factors are indicated. The species and their accession numbers in GenBank (*G. hybrida* (Gh): GhMYB1 (CAD87007), GhMYB1a (CACN01040333), GhMYB10 (CAD87010); *Arabidopsis thaliana* (At): AtMYB11 (NP_191820), AtMYB12 (NP_182268), AtMYB111 (NP_199744), AtTT2 (CAC40021), AtPAP1 (AAG42001), AtPAP2 (AAG42002), AtMYB113 (NP_176811); *Vitis vinifera* (Vv): VvMYBF1 (ACT88298), VvMYBPA1 (CAJ90831), VvMYBA1 (BAD18977), VvMYBA2 (BAF31138.1); *Lotus japonicas* (Lj): LjTT2a (BAG12893); *Zea mays* (Zm): ZmP (P27898), ZmC1 (1613412E); *Gentiana trifloral* (Gt): GtMYBP3 (BAM71801); *Epimedium sagittatum* (Es): EsMYBF1 (ANZ79233); *Picea mariana* (Pm): PmMBF1 (AAA82943); *Malus domestica* (Md): MdMYB10a (ABB84753); *Petunia hybrida* (Ph): PhAN2(AAF6672); *Solanum lycopersicum* (Sl): SlMYB12 (NP_001234401); *Nicotiana tabacum* (Nt): NtAN2 (NP_001312447)) are listed here. **c** Subcellular localization of the GFP:GhMYB1a fusion protein in tobacco leaves. Free GFP served as a control. A DAPI staining assay was conducted to confirm the nuclear localization. Bars = 50 μm

We obtained full-length *GhMYB1*-like cDNA from ray florets in our previous transcriptome analysis of *Gerbera hybrida* cv. Shenzhen No. 5, which has orange petals (GenBank accession number GACN01040333)^30^. Because of the high-protein sequence similarity (98.64%) with that of GhMYB1, which was identified by Elomaa^29^, we designated it GhMYB1a. *GhMYB1a* was predicted to have an open reading frame of 1107 bp, encoding an R2R3-MYB protein comprising 369 amino acids. The protein sequence of GhMYB1a differed from that of GhMYB1 by four amino acids at positions 122, 275, 277, and 306 (Fig. 1a). Phylogenetic analysis showed that GhMYB1a belongs to subgroup 7 (SG7) and clustered with other flavonol MYB regulators together into a flavonol clade (Fig. 1b), suggesting that GhMYB1a probably functions as a regulator of flavonol biosynthesis.

Sequence analysis revealed that GhMYB1a contains the characteristic R2 and R3 MYB DNA-binding domains at the N-terminus (Fig. 1a). Although sequence similarities between MYB proteins are generally restricted to the N-terminus, the SG7 motif (GRTxRSxMK), which is characteristic of flavonol biosynthesis regulators in Arabidopsis and grapevine^14,26^, was also found at the C-terminus of GhMYB1a (Fig. 1a). GhMYB1a also contains the SG7-2 motif ([W/x][L/x]LS) identified previously^26^ in the C-terminal region. In addition, GhMYB1a does not have a [D/E]Lx2[R/K]x3Lx6Lx3R motif, which is responsible for interacting with bHLH proteins^31^.

To verify the localization of GhMYB1a, a construct encoding GhMYB1a fused to green fluorescent protein (GFP) was transformed into tobacco leaves. Strong fluorescence from GFP-GhMYB1a was detected in the nucleus (Fig. 1c), indicating that GhMYB1a localizes to the nucleus. These results suggested that GhMYB1a might function as a transcription factor in regulating flavonol biosynthesis.

### Overexpression of *GhMYB1a* in tobacco and *Gerbera* suppresses anthocyanin accumulation in petals

**Fig. 2:**
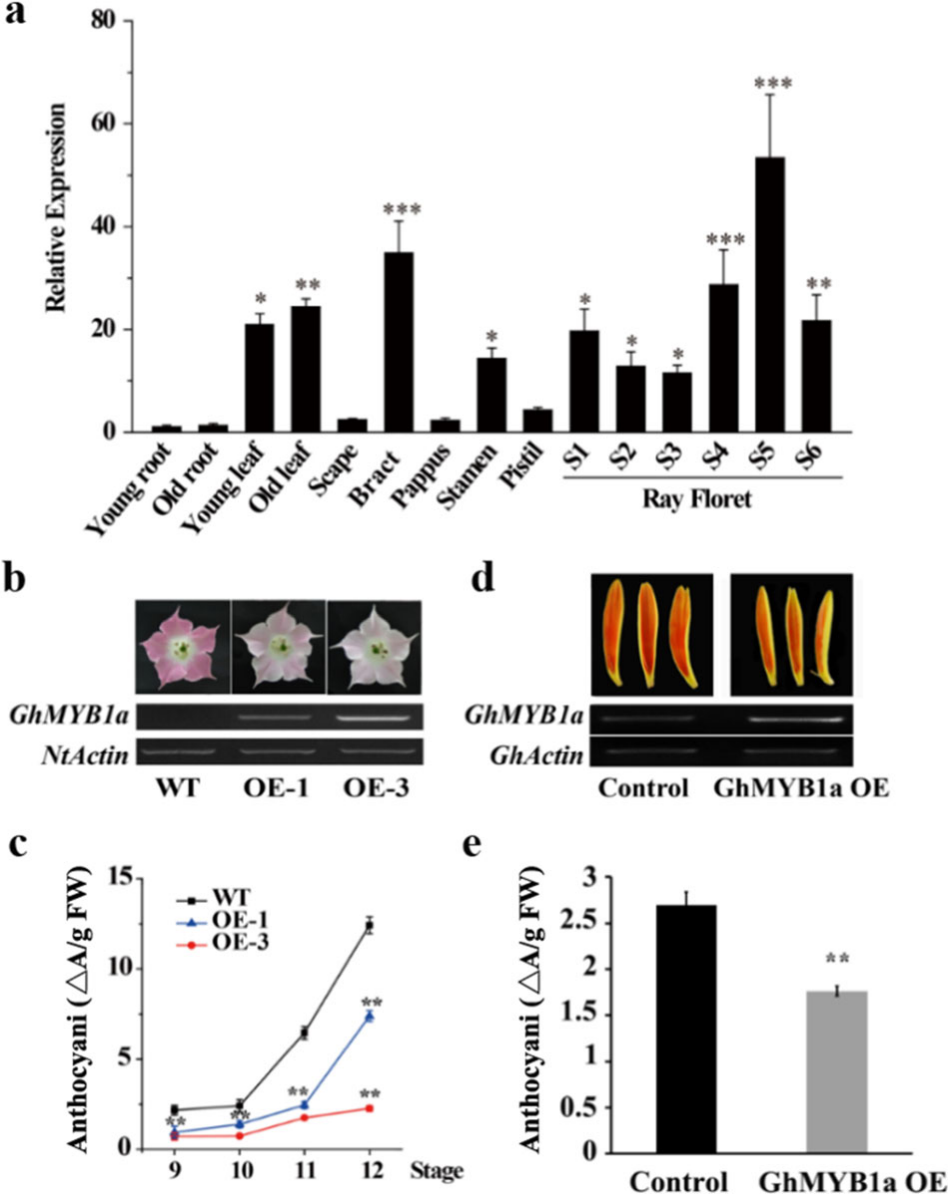
Overexpression of *GhMYB1a* suppresses anthocyanin accumulation. **a** Tissue- and floral development stage-specific expression of *GhMYB1a* in *gerbera*. Scapes, sepals, pappi, stamens, and pistils were harvested from stage 3 (S3) ray florets. The expression in young roots was set to 1. The representative results are shown as the means ± SDs. *P < 0.05, **P < 0.01, ***P < 0.001. **b** Images of stage 12 flowers from WT and transgenic tobacco (upper panes) and their corresponding *GhMYB1a* transcription levels (lower panes); *NtActin* was used as an endogenous control. **c** Anthocyanin accumulation in petals of WT and *GhMYB1a*-overexpressing tobacco plants. The petals were harvested from stage 9, 10, 11, and 12 flowers (*n* = 3). Three independent experiments were performed for each sample. The data are shown as the means ± SDs. **P < 0.01, ***P < 0.001. **d** Transiently expressed *gerbera* petals were generated from stage 3 ray florets (upper panes), and their corresponding *GhMYB1a* transcription in the petals is shown (lower panes). A pCanG vector without a *GhMYB1a* construct was used as a control, and *GhActin* was used as an endogenous control. **e** Anthocyanin content in *GhMYB1a*-overexpressing *gerbera* petals. At least three independent experiments were performed for each sample, and a representative result is shown. More than 25 petals were included in each sample

Reverse transcription quantitative PCR (RT-qPCR) revealed that *GhMYB1a* was highly expressed in *Gerbera* leaves and floral organs, including bracts, stamens, and petals (Fig. 2a). During inflorescence development, transcription of *GhMYB1a* increased gradually and peaked at stage 5^32^ in *Gerbera* cultivar Shenzhen No. 5 (Fig. 2a). However, different expression patterns were observed in other cultivars, e.g., *GhMYB1a* expression peaked at the last stage (S6) in the cultivar Da Tou Fen, which has pink petals, and at stage 4 (S4) in the cultivar Xiang Bin, which has yellow petals (Supplementary Fig. S1a, b).

To characterize the functions of GhMYB1a in regulating petal development, we constructed transgenic tobacco lines overexpressing *GhMYB1a* (*GhMYB1a* OE). Seven independent overexpression (OE) lines were obtained. All seven T_1_ lines exhibited similar phenotypes (data not shown). Two T_2_ lines (OE-1 and OE-3) with different *GhMYB1a* transcript levels were selected for further study (Fig. 2b). The flower color of these two *GhMYB1a OE* lines changed from rosy red (the wild-type (WT) color) to light pink, and the OE-3 line exhibited the lightest coloration (Fig. 2b). Correspondingly, the total anthocyanin content was significantly decreased in the two OE lines (*P* < 0.01) compared with that of the WT (Fig. 2c). As flower growth progressed^33^ (stage 9–12), overexpression of *GhMYB1a* induced stronger suppression of anthocyanin accumulation, especially at stage 12 (*P* < 0.01) (Fig. 2c). Consistent with the observations in transgenic tobacco flowers, transient overexpression of *GhMYB1a* (*GhMYB1a OE*) in *gerbera* petals resulted in dramatic suppression of petal coloration compared to that of the controls (Fig. 2d). Correspondingly, the total anthocyanin content of the *GhMYB1a-*overexpressing *gerbera* petals was significantly lower than that of the control (*P* < 0.01) (Fig. 2e). Taken together, these results indicated that overexpression of *GhMYB1a* results in a significant decrease in anthocyanin accumulation.

### Overexpression of *GhMYB1a* has different effects on the expression of the structural genes involved in anthocyanin biosynthesis

**Fig. 3:**
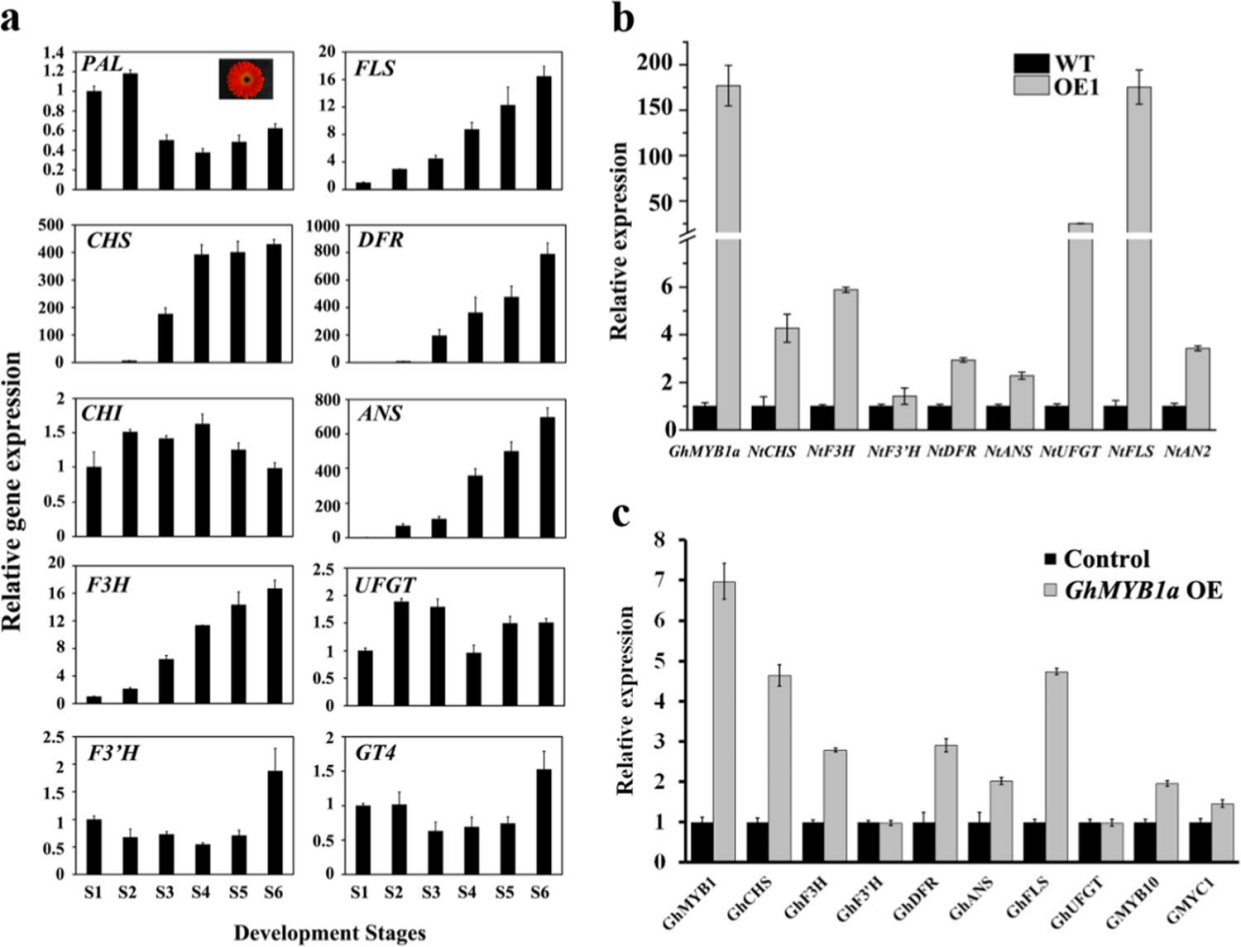
Expression analysis of endogenous flavonoid biosynthetic genes in wild-type and transgenic tobacco and *gerbera* petals. **a** Expression patterns of endogenous flavonoid biosynthetic genes in the petals of the *gerbera* cultivar Shenzhen No. 5 during different developmental stages (S1 to S6). The effects of *GhMYB1a* overexpression on endogenous biosynthetic genes were investigated using RT-qPCR analyses in wild-type (WT) and stage 12 transgenic tobacco flowers **b** and in stage 3 transient overexpression *gerbera* petals, with an empty vector used as a control, in the transient overexpression assays **c**. *Actin* was used as an endogenous control. At least three independent experiments were performed for each sample, and a representative result is shown. The data are the means ± SDs

As a transcription factor, GhMYB1a was hypothesized to regulate the transcription of downstream genes. To investigate whether GhMYB1a affects the accumulation of anthocyanin by regulating the transcription of anthocyanin biosynthetic structural genes, we used RT-qPCR to analyze their expression in the *GhMYB1a* overexpression lines. First, we found that the expression of structural genes varied at different developmental stages in the wild-type plants of different *gerbera* varieties. In cultivar Shenzhen No. 5, the abundance of transcripts of *GhCHS*, *GhF3H*, *GhFLS*, *GhDFR*, and *GhANS* increased gradually, whereas the transcript of *GhPAL* was mildly upregulated at S2 but then decreased at S3 (Fig. 3a). Similar expression patterns of these genes were observed in the cultivar Da Tou Fen, except for the expression of *GhANS*, which peaked at S5 and then slightly decreased at S6, as well as the expression of *GhPAL*, which declined gradually during inflorescence development (Supplementary Fig. S2a). Consistent with its expression pattern in other varieties, the expression of *GhDFR* increased gradually in the cultivar Xiang Bin, whereas the rest of the genes examined showed a biphasic pattern in which the expression increased from the first stage, peaked at S4, and then greatly decreased at S6 (Supplementary Fig. S2b). *GhCHI* and *GhUFGT* were expressed moderately during inflorescence development in the cultivars Shenzhen No. 5 (Fig. 3a) and Da Tou Fen (Supplementary Fig. S2a), whereas their expression decreased significantly from S4 in the cultivar Xiang Bin (Supplementary Fig. S2b). The expression of *GhF3’H* and *GhGT4* mildly increased at S6 in cultivar Shenzhen No. 5 (Fig. 3a) but exhibited different expression patterns in other varieties. The transcript of *GhF3’H* increased throughout the initial stages, peaked at S4, and then slightly decreased in the cultivar Da Tou Fen (Supplementary Fig. S2a), whereas it mildly increased from S2 and then dramatically decreased at S5 in the cultivar Xiang Bin (Supplementary Fig. S2b). Transcripts of *GhGT4* gradually decreased during inflorescence development in the cultivar Da Tou Fen (Supplementary Fig. S2a).

Although the accumulation of anthocyanins in the tobacco *GhMYB1a* OE lines was reduced, the expression of *NtCHS*, *NtF3H*, *NtDFR*, *NtANS*, and *NtUFGT* was enhanced by 4.3-, 5.9-, 2.9-, 2.3-, and 24.9-fold, respectively (Fig. 3b). However, no significant change was detected in the expression of *NtF3’H*, which encodes flavonoid-3′-hydroxylase, which catalyzes the biosynthesis of dihydroquercetin. We also observed a similar result from the *gerbera* transient overexpression petals. The expression of *GhCHS*, *GhF3H*, *GhDFR*, and *GhANS* was also upregulated by 4.6-, 2.8-, 2.9-, and 2.0-fold, respectively (Fig. 3c). Similar to what was observed in the tobacco OE lines, in detached *gerbera* petals overexpressing *GhMYB1a*, the transcript level of *GhF3’H* was also not significantly changed (Fig. 3c). In contrast to what was observed in the tobacco OE lines, *GhUFGT* expression was not significantly changed (Fig. 3c). In addition, the expression of *FNSII*, the gene encoding flavone synthase, was not different between the control and GhMYB1a transient overexpression petals (Supplementary Fig. S3). In addition, the anthocyanin biosynthesis regulator *NtAN2* was significantly increased in the tobacco OE lines (Fig. 3b). In *gerbera* petals overexpressing *GhMYB1a*, the expression of *GhMYB10* was also increased, whereas *GhMYC1* expression was unchanged (Fig. 3c).

### GhMYB1a activates the promoters of flavonol pathway genes

**Fig. 4:**
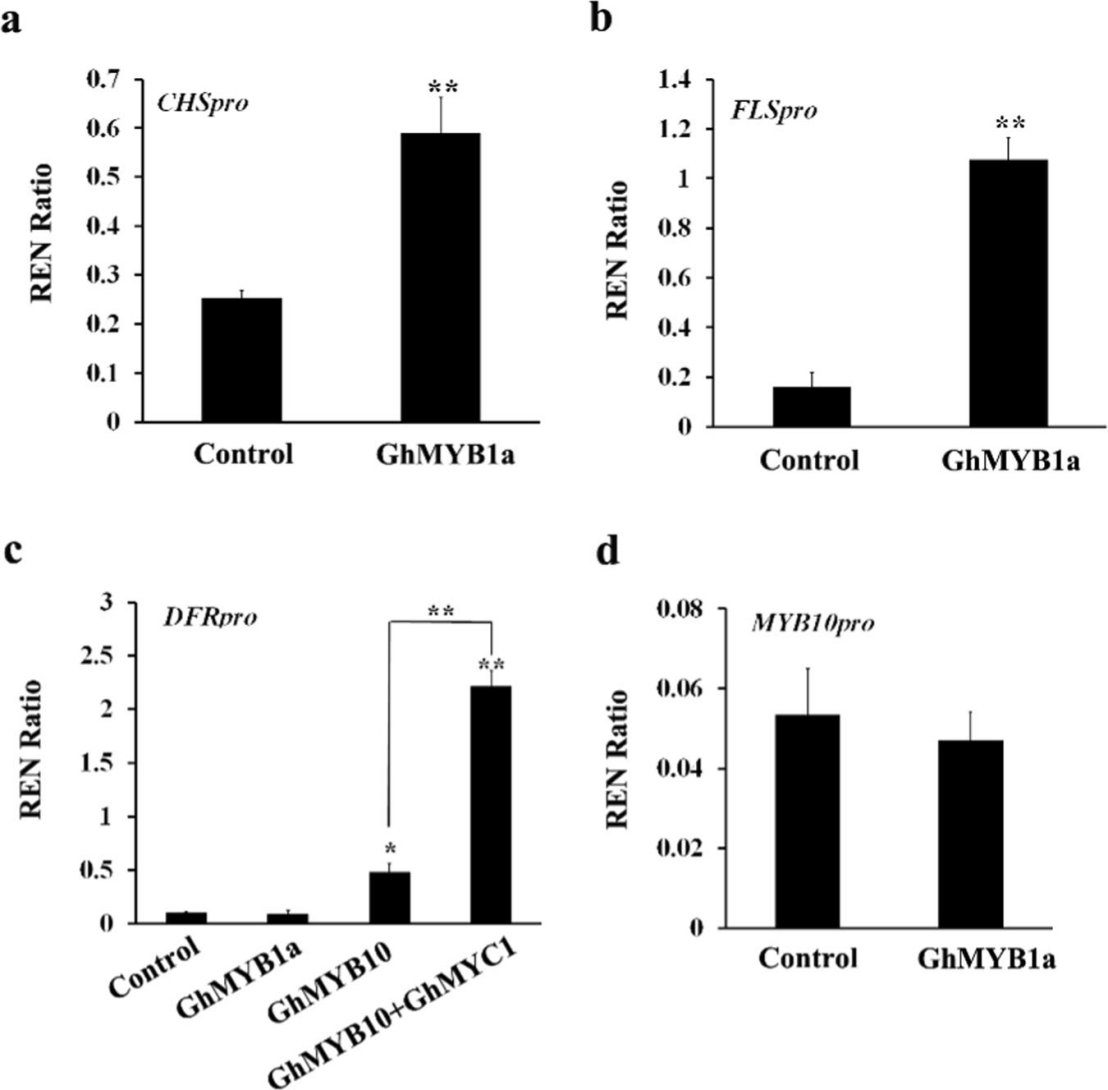
Transcriptional activity assay of *GhMYB1a* against the promoters of flavonoid-related genes of *gerbera* or tobacco. Promoters of *CHS* and *FLS* from tobacco **a**, **b** and DFR and GhMYB10 from *gerbera* **c**–**d** were used for a dual-luciferase assay. GhMYB1a, GhMYB10, and GhMYC1 are from *G. hybrida*. Transformed protoplasts carrying only a promoter-LUC reporter construct without transcription factor-containing effectors were used as controls. The columns represent average values with SD bars from three biological replicates for each treatment

To identify the genes of the flavonoid pathway targeted by GhMYB1a, a dual-luciferase assay in transiently transformed protoplasts of *A. thaliana* was carried out. *CHS* and *F3H*, as early-stage genes of the flavonoid pathway, are involved in the biosynthesis of anthocyanins, proanthocyanidins, and flavonols. *DFR* and *ANS*, which are late-stage genes of the flavonoid pathway, are required for anthocyanin biosynthesis, whereas *FLS*, as a flavonol-specific branch point gene, is required specifically for flavonol biosynthesis. Therefore, the promoters of *CHS*, *DFR*, and *FLS* were chosen as potential targets of GhMYB1a transcription activation. Although we failed to clone the promoter sequences of *CHS* and *FLS* from *gerbera*, the overexpression of *GhMYB1a* resulted in a similar phenotype in both tobacco and *gerbera* petals; therefore, we cloned their promoter sequences from *N. tabacum* (K326). The results showed that GhMYB1 significantly activated the promoters of both *NtCHS* and *NtFLS* but did not activate the promoter of *GhDFR* compared with the corresponding controls, which only consisted of promoters without GhMYB1a. GhMYB10, a regulator of the flavonoid pathway and a positive control, strongly activated the promoter of *GhDFR* and had a stronger effect when combined with GhMYC1 (Fig. 4a–c). In addition, the relative values of transcriptional activation by GhMYB1a of the promoter of *GhMYB10* were approximately equal to those of the empty controls (promoter without GhMYB1a activity) (Fig. 4d), suggesting that GhMYB1a cannot activate the promoters of *GhMYB10*. Taken together, these results suggest that GhMYB1a is a specific regulator of flavonol synthesis, potentially regulating the early-stage genes of the flavonoid pathway, such as *CHS* and *FLS*, cannot directly regulate the late-stage genes such as *DFR*, and is independent of GhMYB10 and GhMYC1 cofactors in regulating the flavonoid pathway.

### Overexpression of *GhMYB1a* accelerates the accumulation of flavonols in tobacco and *gerbera* petals

**Fig. 5:**
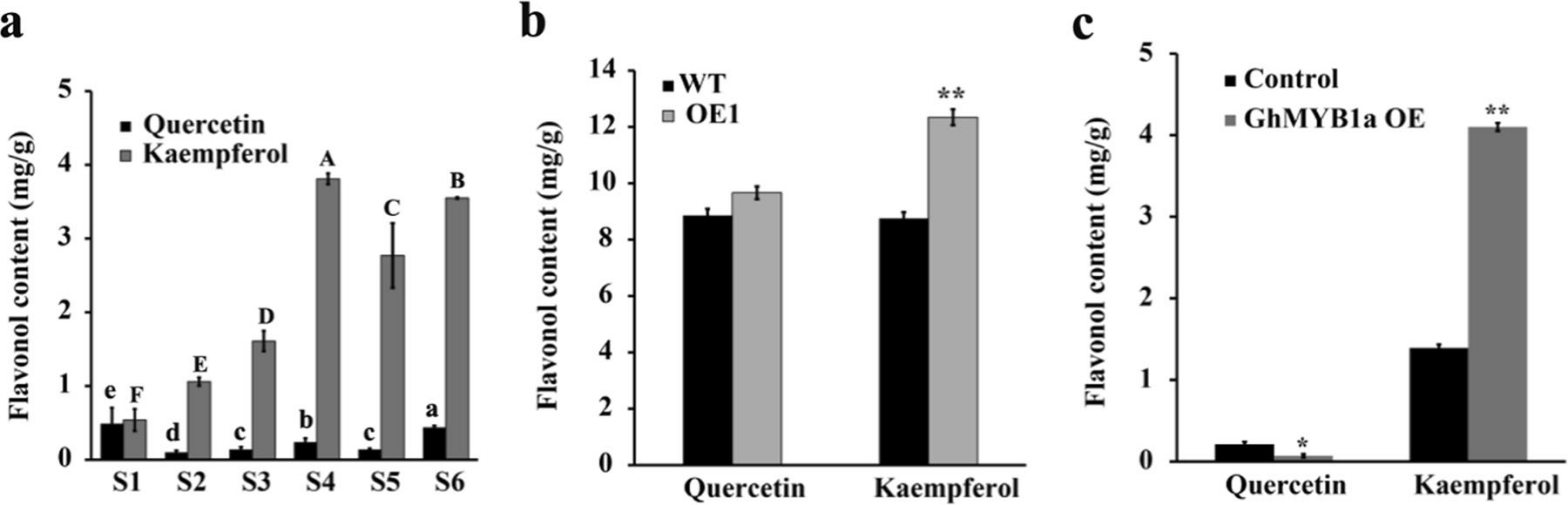
Overexpression of *GhMYB1a* increases flavonol accumulation. **a** Contents of quercetin- and kaempferol-type flavonols in *gerbera* petals during different inflorescence development stages (S1–S6). The letters above the bars indicate significant differences between the respective values (*P* < 0.05). Contents of quercetin and kaempferol in WT and stage 12 *GhMYB1a*-overexpressing tobacco flowers **b** and in stage 3 transient overexpression *gerbera* petals, with an empty vector used as a control, in the transient assays. **c** At least three independent experiments were performed for each sample, and the data are presented as the means ± SDs. *P < 0.05, **P < 0.01, one-way ANOVA

The results of the phylogenetic analysis suggested that GhMYB1a probably functions as a regulator of flavonol biosynthesis. Therefore, we speculated that GhMYB1a promotes flavonol biosynthesis and in turn indirectly affects the accumulation of anthocyanin. To test this hypothesis, we measured the expression of *GhFLS*, which is the critical gene that controls the biosynthesis of specific flavonols, such as kaempferol and quercetin. First, we measured the dynamics of kaempferol and quercetin contents in *gerbera* ray petals during inflorescence development. The content of kaempferol gradually increased from S1 to S4 and was maintained at a high level during the last three stages, S4 to S6, with an approximately five to sevenfold increase over the level at S1 (Fig. 5a). Notably, Pearson’s correlation analysis showed that the expression of *GhMYB1a* was highly correlated with kaempferol content during inflorescence development (Supplementary Table S1). Compared with the kaempferol contents, the quercetin contents were extremely low from S2 to S6 (Fig. 5a). As expected, in petals of the OE tobacco lines and detached *gerbera* petals overexpressing *GhMYB1a*, the expression of *NtFLS* and *GhFLS* was markedly upregulated (Fig. 3b, c). Consistent with this, a significant increase in kaempferol content was observed in the flowers of the overexpression tobacco lines (28%) and in the *gerbera* transient overexpression petals (195%) (Fig. 5b, c). However, no marked change was detected in the quercetin content in the transgenic tobacco flowers, whereas a significant decrease was observed in the transient expression *gerbera* petals (Fig. 5b, c).

### Self-interaction of GhMYB1a in vivo

**Fig. 6:**
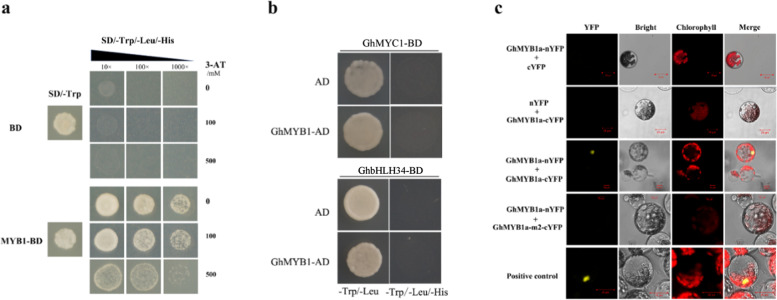
GhMYB1a forms a homodimer in vivo. **a** Yeast two-hybrid assay showing GhMYB1a autoactivation in yeast cells. Transformants were selected on media lacking histidine (His) but including 3-AT at the indicated concentration. **b** Yeast two-hybrid assay showing that GhMYB1a does not interact with GhMYC1 or GhbHLH34. Transformants were selected on media lacking histidine (His). **c** BiFC assay showing that GhMYB1a interacts with itself in *gerbera* mesophyll protoplasts. *YFP* yellow fluorescent protein signal, *Chlorophyll* chloroplast autofluorescent signal, *Bright* protoplasts in light view, *Merge* merged images of YFP chlorophyll, and the bright-field view. The interaction of the bHLH transcription factors AtHBI1-nYFP and AtIBH1-cYFP was used as a positive control. A mutated version GhMYB1a-m2 was used as a negative control

We used a yeast two-hybrid (Y2H) screen to identify partner proteins interacting with GhMYB1a in *gerbera*. A preliminary experiment showed that GAL4 DNA-binding domain (BD)-fused GhMYB1a led to a positive result (Fig. 6a); therefore, we generated GhMYB1a fused to the GAL4 activation domain (AD) and then co-transformed this with GhMYC1, which interacts with GhMYB10 in regulating anthocyanin biosynthesis in *gerbera*^29^, fused to a BD domain. The results showed that GhMYB1a could not interact with GhMYC1 or another bHLH factor, GhbHLH34 (Fig. 6b). Because of the autoactivation of GhMYB1a observed in yeast cells, we used a bimolecular fluorescence complementation (BiFC) assay to examine the GhMYB1a protein interaction. Reciprocal fusions of GhMYB1a with the N- or C-terminal half of YFP (nYFP and cYFP, respectively) were generated and co-transformed into Arabidopsis mesophyll protoplasts in combinations. The mutated version GhMYB1a-m2, which contains the nuclear localization signal but mutated R2R3 regions, was used as a negative control. Strong YFP fluorescence signals were observed in the nucleus when GhMYB1a-nYFP was co-transformed together with GhMYB1a-cYFP. GhMYB1a-m2-cYFP did not interact with GhMYB1a-nYFP, although GhMYB1a-m2 was also located in the nucleus, suggesting that GhMYB1a probably functions as a homodimer in the regulation of flavonol biosynthesis (Fig. 6c).

## Discussion

Amino-acid sequence analysis of GhMYB1a revealed the presence of the highly conserved R2R3 domain at the N-terminus, which is characteristic of MYB transcription factors (Fig. 1a). GhMYB1a shares high-protein sequence similarity with GhMYB1, which was identified by Elomaa et al.^29^. However, the function of GhMYB1 is also not clear in *G. hybrida*. Similar to the MYB protein P from maize and AtMYB11, AtMYB12, and AtMYB111 from Arabidopsis, which function independently of bHLH cofactors^11,23,34^, GhMYB1a does not have the conserved motif for interacting with the bHLH protein (Fig. 1a), suggesting that GhMYB1a functions independently from bHLH transcription factors. GhMYB1a seems to be specific for MYB transcription factors of the flavonol clade (Fig. 1b). In addition, motifs SG7 and SG7-2 have been used to identify MYB transcription factors in plants, which are thought to be involved in regulating flavonol biosynthesis^14,26^ and were also found in the C-terminal region of GhMYB1a. Subcellular localization analysis revealed that GhMYB1a is localized in the nucleus (Fig. 1c), suggesting that GhMYB1a might function as a transcription factor in the nucleus. Taken together, these results suggest that GhMYB1a probably functions as a transcription factor in regulating flavonol biosynthesis in *gerbera*.

In *gerbera* cultivar Terraregina, kaempferol-type flavonols are present mainly in the petals and pappi, whereas quercetin-type flavonols are nearly undetectable in the petals but are dominant in the pappi^35^. Similarly, in cultivar Shenzhen No. 5, kaempferol-type flavonols also predominantly accumulate in petals and peak at the late stages of inflorescence development, whereas low levels of quercetin-type flavonol are detected in the petals (Fig. 5a). The expression pattern of *GhMYB1a* (Fig. 2a) and Pearson’s correlation analysis (Supplementary Table S1) showed that the expression of *GhMYB1a* is significantly correlated with the accumulation of kaempferol during *gerbera* inflorescence development, suggesting that GhMYB1a is possibly involved in the regulation of the kaempferol-type flavonol biosynthetic pathway. Phenotypic analysis of the *GhMYB1a* transgenic lines further confirmed the function of GhMYB1a in the regulation of flavonol accumulation. Transformation of tobacco and transient transfection of *gerbera* petals with the *GhMYB1a* overexpression construct caused a significant increase in flavonol production (Fig. 5b, c). It is worth noting that there were differences in the level of accumulation of the two kinds of flavonols in the transgenic tobacco and *gerbera* petals. Significantly enhanced accumulation of kaempferol-type flavonols was detected in the transgenic tobacco flowers and *gerbera* petals, whereas the accumulation of quercetin-type flavonols was similar to that in the wild type (Fig. 5b, c). These results indicate that *GhMYB1a* is involved in the developmental regulation of kaempferol-type flavonol biosynthesis in *gerbera* petals.

Interestingly, we found that the expression pattern of *GhMYB1a* differed in different *gerbera* cultivars (Supplementary Fig. S1), as did that of the flavonoid biosynthetic genes (Supplementary Fig. S2). This is probably owing to species-specific variations in the types and accumulation levels of flavonoids in *gerbera*, which show tight spatial and temporal regulation^36^. However, whether GhMYB1a is also involved in flavonol biosynthesis in *gerbera* cultivars other than Shenzhen No. 5 remains unknown.

In most plant species, anthocyanins and flavonols are synthesized within the same cell and usually accumulate in the same subcellular location^7^. Thus, potential competition may exist between anthocyanins and flavonols. The overexpression of *GhMYB1a* resulted in an excess accumulation of kaempferol, whereas it strongly reduced petal pigmentation during flower development. Therefore, we further determined the content of anthocyanin in the transgenic lines as well as in the wild type and controls. Compared with those of wild-type tobacco flowers, the anthocyanin contents of the transgenic tobacco flowers were significantly reduced during floral development (Fig. 2c). In addition, the anthocyanin content was significantly reduced in transiently transformed *gerbera* petals (Fig. 2e). The inverse correlation between anthocyanin and flavonol levels in the *GhMYB1a*-overexpressing lines probably reflects competition between these two branches of flavonoid metabolites.

Heterologous expression of subgroup 7 MYB transcription factors in transgenic plants can modulate the expression of flavonoid pathway genes, in particular, by upregulating flavonol pathway genes, resulting in enhanced flavonol biosynthesis and in turn inhibiting the accumulation of anthocyanins^26,27,37,38^. Overexpression of *GhMYB1a* in tobacco and *gerbera* petals also resulted in increased expression of flavonol pathway genes and an increased accumulation of flavonols (Fig. 3). Previous studies have shown that flavonol pathway genes, including *PAL*, *CHS*, *CHI*, *F3H*, and *FLS*, which lead to flavonol biosynthesis, can be generally increased in transgenic tobacco by overexpressing flavonol-specific MYB transcription factors^25,39,40^. In *GhMYB1a*-overexpressing transgenic tobacco lines, the expression of three genes, *NtCHS*, *NtF3H*, and *NtFLS*, was more strongly upregulated than most of the other upregulated genes of the flavonoid pathway, whereas the expression of *F3’H* was unchanged (Fig. 3b).

During inflorescence development in *gerbera* cultivar Shenzhen No. 5, the dynamic expression of *GhCHS*, *GhF3H*, and *GhFLS* was consistent with the accumulation of flavonols, particularly kaempferol (Figs. 3a and 5a). Moreover, the transcription activity assay showed that GhMYB1a significantly activates the promoters of *NtCHS* and *NtFLS* (Fig. 4a, b). The expression of two late-stage anthocyanin biosynthetic pathway genes, *DFR* and *ANS*, was previously reported to be not significantly modulated^37^ or downregulated^25,27,38^ in transgenic tobacco lines ectopically expressing these flavonol-specific MYB transcription factors. However, our results showed that the expression levels of *DFR*, *ANS*, and *UFGT* markedly increased in tobacco and *gerbera* transgenic lines (Fig. 3b, c). This is probably owing to the upregulation of *NtAN2* in transgenic tobacco or *GhMYB10* in *gerbera* (Fig. 3b, c). However, the transcription activation assay showed that GhMYB1a cannot directly activate the promoter of *GhDFR* or *GhMYB10* (Fig. 4c, d), indicating that the upregulation of late-stage genes results from indirect regulation by the GhMYB1a transcription factor. In addition, the expression of *FNSII*, the gene encoding flavone synthase, was not altered between the control petals and *GhMYB1a* transient overexpression petals (Supplementary Fig. S3). Taken together, these results indicate that GhMYB1a affects the accumulation of flavonols mainly through directly regulating the expression of the early-stage genes (*CHS*, *F3H*, *FLS*) of the flavonoid pathway but not the late-stage genes (*F3’H*, *DFR*, *ANS*). These results are consistent with those of previous studies of other subgroup 7 MYB transcription factors.

These target genes for *GhMYB1a* regulation are indispensable for flavonol biosynthesis. Blocking target genes that encode enzymes at metabolic branch points may allow the control of metabolic flux^36^. DFR is the first enzyme in the anthocyanin branch of flavonoids and competes with FLS for substrates^41^. A *gerbera* cultivar with white petals, Ivory, accumulates higher kaempferol than does its wild-type cultivar Estelle owing to a mutation in *DFR*. The expression of *DFR* increased in the *GhMYB1a*-overexpressing lines, whereas the expression of *F3’H* was not changed. As *F3’H* is the key target gene for initiating the cyanidin pathway in *gerbera* tissue^42^, a relatively low production of *F3’H* creates an imbalance in the flux through the pathway, leading to a reduction in dihydroquercetin (DHQ), which in turn inhibits the accumulation of quercetin as well as cyanidin. In summary, these results suggest that the combination of the reduction in DHQ, the accumulation of dihydrokaempferol, and the excess amount of *FLS* transcripts results in the increased accumulation of kaempferol and the reduction in anthocyanin in *GhMYB1a*-overexpressing lines.

The mechanism of R2R3-MYB transcription factors in the regulation of anthocyanin biosynthesis has been widely studied. Most R2R3-MYB transcription factors depend upon cofactors, such as bHLH and/or WDR proteins^14,18,19^, whereas the other R2R3-MYB transcription factors regulating flavonol biosynthesis are bHLH independent. In *gerbera*, GhMYB10, an activator of pigmentation accumulation in *gerbera* petals, strongly interacts with GhMYC1 and enhances anthocyanin biosynthesis^29^. We used a yeast two-hybrid assay to further investigate whether GhMYB1a affects GhMYB10 or GhMYC1 at the protein level. The results showed that GhMYB1a does not interact with bHLH factors such as GhMYC1 and bHLH34 (Fig. 6b), which is probably owing to the absence of the bHLH interaction motif in GhMYB1a. However, GhMYB1a can form a homodimer in *gerbera* protoplasts (Fig. 6c). These results suggest that the mechanism of the flavonol-specific transcription factor GhMYB1a is different from that of the anthocyanin regulators.

Our findings not only provide further understanding of the regulation of the flavonol biosynthetic pathway in *gerbera* but also highlight a potential regulator that could be used for genetic manipulation to improve the accumulation of pigmentation. Additional studies on how anthocyanin pathway regulators such as GhMYB10 modulate flavonol pathway regulators such as GhMYB1a should increase our understanding of the mechanism underlying the regulation of anthocyanin and flavonol accumulation.

## Materials and methods

### Plant materials and growth conditions

Seedlings of the *gerbera* cultivars Shenzhen No. 5, Da Tou Fen, and Xiang Bin were planted in the greenhouse at the Zengcheng (Guangzhou, Guangdong, China) Ornamental Center, where the temperature was 26/18 °C (day/night) and the relative humidity was 65~80%. For in vitro culture, as described previously^32,42,43^, ray floret petals (at stage 3^32^) were removed from the inflorescences of the plants and then gently placed on filter paper soaked with a 1% sucrose solution.

*N. tabacum* (K326) was used for the generation of transgenic plants using the *Agrobacterium*-mediated leaf disc transformation method as described previously^44^. The tobacco plants were grown in a growth chamber at a temperature of 25 °C, a light intensity of 10,000 lx and a humidity of 65~80% and then ultimately transferred to the greenhouse.

*A. thaliana* (Columbia) seeds were sterilized with 1% NaClO_3_, plated on Murashige and Skoog media, chilled at 4 °C for 3 d, and transferred to the greenhouse under 24 h of light at 22 °C.

### Isolation of *GhMYB1a* from *gerbera*

Total RNA was isolated from *gerbera* ray florets using a plant total RNA isolation kit (Huayueyang Biotechnology, Beijing). The experiment was performed according to the manufacturer’s instructions, followed by reverse transcription into cDNA using a PrimeScript RT Reagent Kit with gDNA Eraser (Takara, Japan). Amplification of full-length *GhMYB1a* cDNA with gene-specific primers (forward primer: 5′-AGTGTAAGTATGGGAAGAGCG-3′; reverse primer: 5′-ATTAAGAAAGAAGCCATGCAACC-3′) was performed by PCR, after which the sequence was generated. The generated sequence was checked by BLASTX and BLASTp against GenBank, and the sequences of the homologs were retrieved to perform an alignment analysis.

### Generation of *GhMYB1a*-overexpressing tobacco

A *CaMV35S::GhMYB1a* construct was generated by inserting the complete open reading frame of the *GhMYB1a* coding sequence into a pCanG vector, which was then transferred into an LBA4404 *Agrobacterium* strain. *Agrobacterium*-mediated transformation of tobacco plants was subsequently carried out by the method described previously^44^. Carbenicillin (200 mg/L) and kanamycin (50 mg/L) were used for selecting the transgenic tobacco lines. After rooting and acclimation, transgenic plants were transferred to the greenhouse and grown until flowering. RT-PCR was used to verify the insertion of *GhMYB1a* in the transgenic tobacco plants. T_2_ transgenic plants were used for further analysis.

### Transient overexpression of *GhMYB1a* in *gerbera* petals

Transient overexpression of *GhMYB1a* in *gerbera* petals followed the method described previously^45^. The *CaMV35S::GhMYB1a* construct and the empty vector without the *GhMYB1a* insert were transferred into *A. tumefaciens* strain C58C1 harboring the Ti plasmid pGV2260, followed by transformation of in vitro-cultured *gerbera* petals (stage 3) using the vacuum penetration method described by Tang et al. (2017). The empty vector without the *GhMYB1a* insert was used as a control. In brief, petals of ray florets were detached from inflorescences and immersed in an *A. tumefaciens* strain C58C1 culture solution, followed by vacuum penetration for 10 min. The petals were rinsed with sterile water and then cultured in vitro at 8 °C in continuous darkness for 3 d. The petals were subsequently transferred to culture at 25 °C in the light (10,000 lx) for further analysis.

### Subcellular localization assay

The full-length cDNA sequence of GhMYB1a was inserted into a modified pCanG vector, resulting in CaMV35S::GFP:GhMYB1a fusion constructs, which were subsequently inserted into *A. tumefaciens* GV3101 by the electroporation method. A pCanG vector containing CaMV35S::GFP was used as a control. *Agrobacterium* was cultured on YEB agar supplemented with selection antibiotics and then incubated at 28 °C for 2~3 d. The confluent Agrobacterium containing the target vector was resuspended in infiltration buffer (10 mM MgCl_2_, 10 mM MES (pH 5.6), 200 μM acetosyringone) to an OD_600_ of 0.4 and incubated at room temperature without shaking for 2 h before infiltration. Approximately 500 μl of the Agrobacterium mixture was then infiltrated into 3–4 young leaves of each plant, with at least two points for each leaf. The subcellular localization assay was performed 3 d after inoculation. Confocal images were taken by using a Zeiss LSM 710 laser scanning microscope (Zeiss, German).

### Dual-luciferase reporter (DLR) assay

Transcriptional activity of the GhMYB1a transcription factor on the promoters of flavonoid biosynthetic genes was assessed using a DLR assay of transiently transformed Arabidopsis protoplasts. Protoplast isolation from Arabidopsis leaves and purification were performed as previously described^46^. The promoter sequences of *GhDFR* (the 1281 bp upstream region from the ATG start site) and *GhMYB10* (the 455 bp upstream region from the ATG start site) genes were isolated from genomic DNA of *G. hybrida* by using a genome walking kit (TaKaRa, Japan). The promoter sequences of *NtCHS* (the 1268 bp upstream region from the ATG start site) and *NtFLS* (the 1275 bp upstream region from the ATG start site) genes were amplified from the *N. tabacum* (K326) genome. All the promoters were subcloned into a pGreenII 0800-LUC transient expression reporter vector, which contains a CaMV35S promoter-REN cassette and the promoterless LUC cassette. Similarly, the coding regions of GhMYB1a, GhMYB10, and GhMYC1 were subcloned into a pGreenII BSK transient expression effector vector, which contains a CaMV35S promoter-MCS-CaMV terminator cassette. All the primers used for reporter and effector constructions are listed in Supplementary Table S2.

All the reporter and effector constructs were transformed into *Arabidopsis* protoplasts with the indicated combinations, as shown in Fig. 4. In brief, 5 μg of reporter and 5 μg of effector plasmid DNA were combined and mixed with 100 μl of protoplasts, followed by the addition of an equal volume of PEG/Ca solution (40% PEG 4000, 0.2 M mannitol, 0.1 M CaCl_2_), and then incubated at room temperature for 6 min. After 3~4 washes with W5 solution (5 mM glucose, 1.5 mM MES (pH 5.7), 154 mM NaCl, 125 mM CaCl_2_, 5 mM KCl), the transformed protoplasts were resuspended in W5 solution at a final concentration of 4 × 10^6^ cells/mL and incubated for 20 h in the dark. Dual-luciferase assays of transiently transformed *Arabidopsis* protoplasts was carried out by using a dual-luciferase reporter assay system (Promega, USA) according to the manufacturer’s instructions. Twenty microliters of crude extract was measured in 100 μl of luciferase assay buffer. Another 100 μl of Stop and Glow buffer was then added. Two buffers were autoinjected for chemiluminescence measurements. Luminescence units were measured using a Synergy microplate reader (BioTek, USA) with a 2 s delay and a 10 s integrated measurement. Activity data were expressed as the ratio of LUC activity to REN activity. Blank controls were run with only the promoter-LUC reporter construct (no transcription factor). In some cases, positive controls were run using GhMYB10 and GhMYC1 with known activity.

### Real-time quantitative PCR (qPCR)

Total RNA was isolated from tobacco (K326) flowers at stage 12 or different tissues of *gerbera* by using a plant total RNA isolation kit (Promega, USA) according to the manufacturer’s instructions, followed by reverse transcription by using a PrimeScript RT Reagent Kit with gDNA Eraser (Takara, Japan). SYBR Premix Ex Taq (Japan) was used for real-time quantitative PCR according to the manufacturer’s instructions. The amount of starting cDNA was adjusted according to the Ct value of the endogenous gene (18~20 cycles). Gene expression in the *gerbera* samples was normalized against the expression of the *GhActin* (AJ763915) gene as previously described^29^; for tobacco, against that of *NtActin* (AB158612). The primers used are listed in Supplementary Table S2.

### Determination of anthocyanin content

The total anthocyanin content was determined as described previously^47^. In brief, ~50 mg of petals was cut into pieces, followed by soaking overnight in the dark in 1 mL of 1% (v/v) HCl/methanol, after which the mixture was centrifuged at 10,000 × *g* in a microcentrifuge for 8 min. Light absorption of the supernatant was measured at 530 nm and 657 nm by using a UV-visible spectrophotometer UV-7500 (Shimadzu, Japan). The total anthocyanin content was calculated as described previously^49^. Three replications were included per sample, and the data are shown as the means ± SDs.

### Flavonoid extraction

The method of extracting flavonoids was described by Chen et al.^48,49^, with some modifications. Approximately 100 mg of tobacco petal powder was extracted by 1 m of 0.2% formic acid-methanol. The supernatant was collected after 15 s of vibration and then sonicated for 20 min by using a KQ-500DE ultrasonic cleaner (Jiangsu, China), after which it was centrifuged at 12,000 rpm for 10 min. The above steps were repeated at least three times until the sediment became transparent. All the supernatants were collected, combined into one tube and then supplemented with up to 3 m of 0.2% formic acid-methanol. After centrifugation, the extraction was filtered through a 0.22 μm Millipore membrane. The clear supernatant was then analyzed by ultra performance liquid chromatography–tandem mass spectrometer (UPLC–MS/MS). Each sample was repeated three times.

### UPLC–MS/MS system and conditions

A 10 cm × 2.1 mm Waters Acquity 1.7 μm BEH C18 column (Waters, Milford, MA, USA) was used for chromatographic separation. A Xevo TQ-MS triple-quadrupole mass spectrometer (Waters, Milford, MA, USA) and an Acquity Ultra Performance Liquid Chromatograph (UPLC I-CLASS, Waters) were used for UPLC–MS/MS analysis to measure the flavonoid contents. Aqueous 10% formic acid was used as eluent A, and absolute acetonitrile was used as eluent B. The following gradient elution protocol was performed as described previously^49^. The system control and data processing were performed by using analytical software (MassLynx, version 4.1).

### Quantitative and qualitative analysis of flavonols

Quercetin 3-O-α-l-rhamnopyranosyl-(1→6)-β-d-glucopyranoside (rutin) is the standard for the semiquantitative analysis of flavonols. Solutions of six different concentrations of rutin were prepared with 0.2% formic acid-methanol (v/v) and then measured with a wavelength of 350 nm using UPLC-PDA. The regression equation was *Y* = 89,961X (*R*_2_ = 0.9958), showing good linearity between the concentration and the peak area. The relative content of total flavonols in each sample is presented in the form of standard milligrams per 100 g DW.

### Yeast two-hybrid assay

The full-length cDNA of *GhMYB1a*, *GhMYC1*, and *GhbHLH34* was fused to pGADT7 (AD) and pGBKT7 (Clontech). The constructs were co-transformed into yeast strain AH109 (Clontech). Synthetic drop-out media lacking tryptophan (SD/-Leu/-Trp) was used for selecting the transformed yeast strains at 28 °C for 3 d, which were then transferred and streaked onto synthetic drop-out media lacking tryptophan and histidine (SD/-Lue/-Trp/-His). 3-Amino-1,2,4-triazole (3-AT) (Sigma, USA) was added to the SD/-Lue/-Trp/-His media for the autoactivity assay of GhMYB1a.

### BiFC analysis

The full-length cDNA of *GhMYB1a* was fused to the N-terminal region of the YFP-coding sequence, giving rise to the plasmid pSAT6-n (1–174) EYFP-C1-GhMYB1a, and the full-length cDNA of *GhMYB1a* was fused to the C-terminal region of the YFP-coding sequence, giving rise to the plasmid pSAT6-cEYFP-GhMYB1a. The pSAT6-n (1-174) EYFP-C1-GhMYB1a and pSAT6-cEYFP-C1-GhMYB1a plasmids were subsequently transformed together into *Arabidopsis* mesophyll protoplasts. The protoplasts were then incubated in the dark at 23 °C for 22 h. An LSM510 Meta Confocal Laser Scanning Microscope (Zeiss, German) was used to detect the fluorescence signals in the transfected protoplasts.

### Statistical analyses

SPSS software was used for statistical analysis. The data are expressed as the means ± SDs. One-way analysis of variance was used to test statistical significance.

## Supplementary information

Supplementary information
