# Supplementary material for: The R2R3-MYB transcription factor GhMYB1a regulates flavonol and anthocyanin accumulation in Gerbera hybrida
Source: Hortic Res. 2020 May 20;7:78. doi: 10.1038/s41438-020-0296-2 (PMC7237480; doi:10.1038/s41438-020-0296-2)
Supplement: Supplementary file 1 — Supplementary information [file 41438_2020_296_MOESM1_ESM.docx]

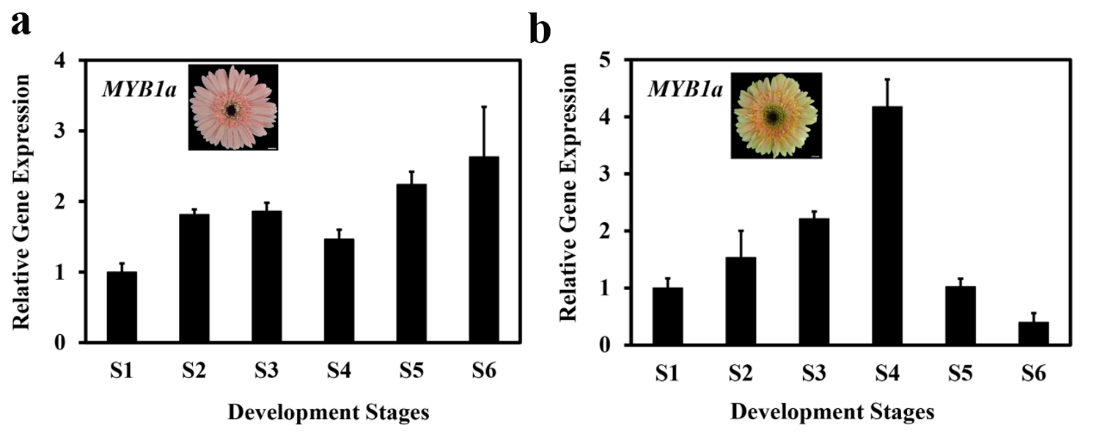


**Supplementary Figure S1. Expression patterns of *GhMYB1a* during different developmental stages (S1 to S6) in diverse gerbera varieties.** (a) Cultivar Da Tou Fen, with pink petals; (b) cultivar Xiang Bin with yellow petals.


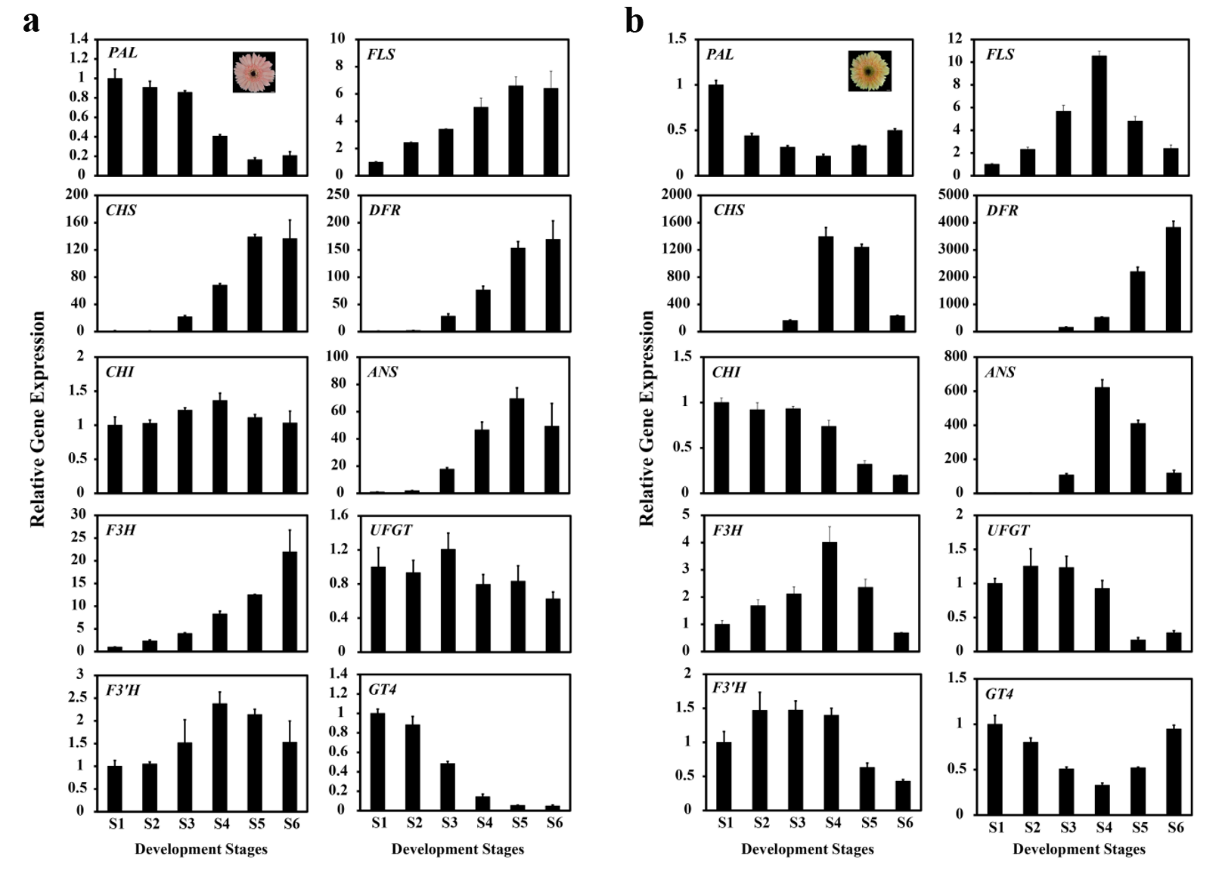


**Supplementary Figure S2. Expression patterns of endogenous anthocyanin biosynthetic genes during different developmental stages (S1 to S6) in diverse varieties.** (a) Cultivar Da Tou Fen, with pink petals; (b) cultivar Xiang Bin, with yellow petals.


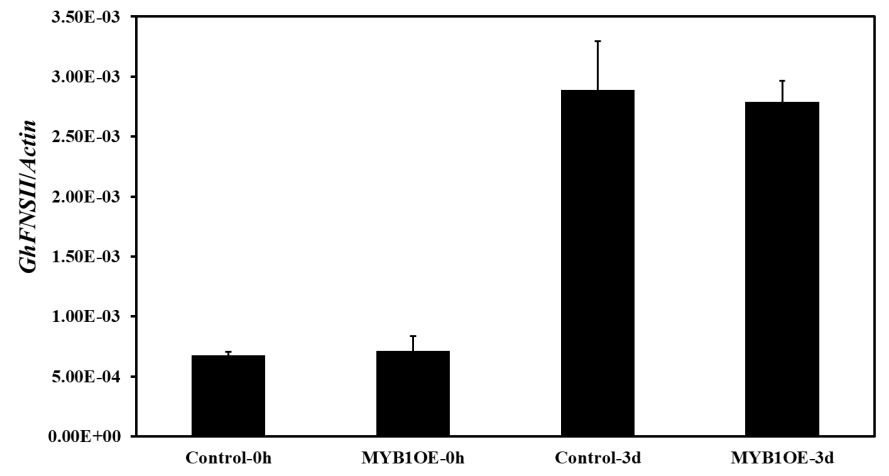


**Supplementary Figure S3.** Expression patterns of the flavone synthase gene *FNSII* in stage 3 transient overexpression gerbera petals, with an empty vector used as a control, in transient overexpression assays. *Actin* was used as an endogenous control.

| **Supplementary Table S1.** Analysis of correlations between *GhMYB1a* expression and kaempferol content | | | |
| --- | --- | --- | --- |
|  | | GhMYB1a | Kaempferol |
| GhMYB1a | Pearson correlation | 1 | 0.585* |
|  | Sig. (2-tailed) |  | 0.046 |
|  | N | 12 | 12 |
| Kaempferol | Pearson correlation | 0.585***** | 1 |
|  | Sig. (2-tailed) | 0.046 |  |
|  | N | 12 | 17 |
| *****: Correlations are significant at 0.05 level (2-tailed) | | | |

**Table S2. Primers used in qRT-PCR and DLR assays**

| **Gene name** | **Accession number** | **Forward primer (5'-3')** | **Reverse primer (5'-3')** |
| --- | --- | --- | --- |
| *NtActin* | AB158612 | GGTTGGATCTTGCTGGTCGT | GCAGTCTCCAACTCTTGCTCA |
| *NtPAL* | AB289452 | AAGAAGCGTTCCGTGTTGCT | GGACCATTGAGGCCAAACCA |
| *NtC4H* | AB236952 | GCTGACTAACAACCCTGCTACCTG | ATTGCCATTAGCCTCAACGTGCTT |
| *NtCHS* | AF311783 | GCTCTTACTTGTTGACGCACG | CGTGATAAGTGTCTATGGGGATTGT |
| *NtCHI* | KJ730247 | TTTTGGGATGATGGTGCGGT | CGTTCTTCCCACCAGTGAGTT |
| *NtF3H* | NM_001325083 | ATGAGCGTCCAAAAGTGGCT | GCCCCAATCTTCACATGCCT |
| *NtF3'H* | AB289449 | TGGAATCCGACCTAGCCCAA | CGGATGCAATTCTAGGGAGGG |
| *NtDFR* | EF421430 | CTGGAGCGACTTGGACTTCAT | CAGCGGCGGTATGATGCTA |
| *NtANS* | AB723683 | CTACCCCAAATGTCCCCAACC | GCCGTTACCCACTGTCCTTC |
| *NtUFGT* | GQ395697 | AGGAAGCCATGCAACTCCTTT | TGAGGTGTTGGATTGTGAAGTGT |
| *NtGT4* | AB176522 | TTCAGTTCAACAATTACTCGTGCCAT | GAAGGAAGCATGTCAATATTTTCGCAAC |
| *NtFLS* | AB289451 | GCTGCGAGAAGTTGTGGAGA | CCTTGGGCATGGTGGGTAAT |
| *NtANR* | AM791704 | AAGATGTTTGTCGCGCCCATA | TTTGCTAGCTCCGGAACACTG |
| *NtAN2* | FJ472651 | TGGAAGGACGGCAAACGATG | GGCCGAGGTCTGAATATGGTG |
| *GhMYB1a* | GACN01040333 | GGCCATGGGATTGGAAATGGA | GTCGTCGTTGTGCTCTCCC |
| *GhPAL* | GACN01001025 | AGAAGGACTTGCTCCGTGTG | AGACTTGTCGGAGCTTCTGC |
| *GhF3’H* | GACN01022724 | CACCCGTCAACCCCACTATC | TCTCGAGAAATGGCCCACAC |
| *GhFLS* | GACN01029930 | AAAGGCGCAATCCCAAATGAC | TGGCTCAGATCGATCACTGG |
| *GhDFR* | GACN01028851 | TTTGGCTGAAAAAGCTGCGT | AACGAGACTTGGTGGGAACG |
| *GhANS* | GACN01028966 | GCATCCACCAGATCCCGAAA | GATCAACGGTGGGGACTTGT |
| *GhUFGT* | GACN01014730 | ACGGTTTTCACGACGATTGC | ACCCAGGTGGTGGTTTCTTC |
| *GhGT4* | GDBL01056407 | TGGATGCTTCACGTTCCTGT | CAATCCGGTCTGACAAGCCA |
| *GhMYB10* | AJ554700 | CACAACCTCGTCAATGGGGA | AAGTCGACATTGACGGCCAT |
| *GhMYC1* | GACN01001173 | GAGAATGGGTCGAGTCTGGC | TCCCTCTAGACGGGACCAAT |
| *GhFNII* | GDBL01044943 | AGCCGGCACCTTGTTGTTTG | ACACCAGGACACCCTCTCCT |
| *NtCHSpro* | AF311783 | TCCGCTTTGACATGCAACTAAT | TTTCGCCGGAAAAAATGGT |
| *NtFLSpro* | AB289451 | TCGGTTGGATATCGACCCG | TTTTCTTCTTAAGACCCTTAATACACG |
| *GhDFRpro* | GACN01028851 | GGAGAAGGTGTTTGGGGAAAG | GTTTTATTTGGTGGGTATTAATTGTTTT |
| *GhMYB10pro* | AJ554700 | AGTAGCAGAGAGGGAGGTGTATTTAAC | TCTTGTTCGTTTGTACTCACTGCA |
| *GhMYB1a-PBS* | GACN01040333 | ATGGGAAGAGCGCCATGC | TTAAGAAAGAAGCCATGCAACCA |
| *GhMYC1-PBS* | GACN01001173 | ATGGAGAACTTGAGGCCAAAAC | TCAATACCTACCGATGACTCTCTGA |
| *GhMYB10-PBS* | AJ554700 | ATGGGTGCTGAGGCACGTT | TTAATTTAAAAGGTCCCACACAACC |
